# Comparison of optic nerve morphology in eyes with glaucoma and eyes with non-arteritic anterior ischemic optic neuropathy by Fourier domain optical coherence tomography

**DOI:** 10.3892/etm.2013.1115

**Published:** 2013-05-15

**Authors:** YUXIN YANG, HAITAO ZHANG, YITAO YAN, YANLING GUI, TIECHUI ZHU

**Affiliations:** 1 Departments of Ophthalmology, The First Affiliated Hospital of Xinxiang Medical University, Weihui, Henan 453100, P.R. China; 2 Nursing, The First Affiliated Hospital of Xinxiang Medical University, Weihui, Henan 453100, P.R. China; 3 Nephrology, The First Affiliated Hospital of Xinxiang Medical University, Weihui, Henan 453100, P.R. China

**Keywords:** optic disc, retinal nerve fiber layer, glaucoma, ischemic optic neuropathy, optic coherence tomography

## Abstract

The aim of this study was to compare the optic nerve head (ONH) and peripapillary retinal nerve fiber layer (RNFL) thickness in eyes with glaucoma and non-arteritic anterior ischemic neuropathy (NAION) by Fourier domain optical coherence tomography (FDOCT), and to evaluate the diagnostic capability of FDOCT in glaucoma and NAION. This study included 26 eyes with glaucoma (36.6%), 15 eyes with NAION (21.1%) and 30 eyes of normal subjects (42.3%). Those with the following conditions were excluded; a visual field defect greater than one hemifield, spherical equivalent (SE) more than ±6 D, or the onset of NAION within 6 months. FDOCT was used to analyze the characteristics of ONH and RNFL thickness. Among the three groups of subjects, glaucomatous eyes had the largest cup area and cup volume, and the smallest rim area, rim volume and disc volume (P<0.05). NAION eyes had the smallest cup area and cup volume (P<0.05), but their rim area, rim volume and disc volume were comparable to those of control eyes (P>0.05). The cup-to-disc (C/D) ratio was increased in glaucomatous eyes but reduced in NAION eyes compared with control eyes. Glaucomatous eyes had the greatest loss of RNFL thickness in the temporal upper (TU), superior temporal (ST) and temporal lower (TL) regions (P<0.05), whereas NAION eyes had the smallest RNFL thickness in the superior nasal (SN) and nasal upper (NU) regions (P<0.05). The areas under the receiver operator characteristic curve (AROCs) of the temporal, superior and inferior RNFL in glaucomatous eyes were greater compared with that of the disc area (P<0.05). In addition, the AROCs of the temporal, superior and inferior RNFL were higher compared with that of nasal RNFL (P<0.05). The AROCs of all parameters for NAION were not significantly different, with the exception of superior, nasal superior and inferior temporal RNFL (P<0.05). In conclusion, FDOCT is able to detect quantitative differences in the optic disc morphology and RNFL thickness between glaucomatous and NAION eyes. These differences may provide new insights into the clinical characteristics and diagnosis of the two diseases.

## Introduction

Glaucoma is an eye disease manifested by the apoptosis of retinal ganglion cells (RGCs) and loss of the retinal nerve fiber layer (RNFL). These pathological changes lead to progressive expansion of the optic cup relative to the size of the optic disc and the characteristic pattern of visual field loss. Non-arteritic anterior ischemic neuropathy (NAION), another optic nerve disease, may also cause degeneration of RGCs after the acute phase. Therefore, similarities in the morphology of the optic nerve, such as the expansion of the optic cup and reduction of RNFL thickness, are often observed in NAION eyes and glaucomatous eyes (1). Previous studies identified no difference in the size of the optic disc between NAION and glaucomatous eyes, but the cup-to-disc (C/D) ratio of NAION eyes was reported to be lower than that of normal or glaucomatous eyes (2,3). In those studies, special training was required for the examiners to determine the exact boundary of the optic disc by using traditional methods (e.g. stereoscopic fundus photography and Heidelberg retina tomography), which lead to human error. Therefore, new tools that are more objective and accurate will be useful for investigating the morphology of the optic nerve in glaucomatous and NAION eyes.

Optical coherent tomography (OCT) is a non-invasive, rapid and reliable technique for imaging biological tissues. It has been widely used in the diagnosis of optic neuropathy, including glaucoma. Fourier domain OCT (FDOCT) is a new generation of OCT system which has higher resolution (5 *μ*m) and faster scanning speed (26,000 A-scan/sec) compared with the previous generation. It has been shown that FDOCT has high repeatability for the measurement of RNFL thickness and optic disc parameters (4). The high sensitivity and specificity make FDOCT a valuable tool for detecting pathological changes of the optic nerve, including glaucoma and NAION (5). Although comparisons of the optic nerve morphology between glaucoma and NAION have previously been reported (6–9), to the best of our knowledge, no studies have analyzed the characteristics of the optic disc and RNFL with FDOCT. In the current study, we measured the parameters of the optic disc and RNFL thickness in glaucomatous and NAION eyes and described the clinical characteristics of these two diseases.

## Subjects and methods

### Clinical data

Adult patients (age ≥40) diagnosed in The First Affiliated Hospital of Xinxiang Medical University (Weihui, China) between October 2011 and September 2012, were considered for inclusion. One eye was randomly chosen for analysis. Subjects were divided into three groups: the glaucoma, NAION and normal control groups. Subjects that presented with the following conditions were excluded from analysis: a visual field defect larger than one hemifield, spherical equivalent (SE) more than ±6 D, or the onset of NAION within 6 months.

The glaucoma group included eyes with: i) characteristic changes in rim morphology and RNFL thickness; ii) characteristic visual field defects (e.g. nasal steps, Bjerrum scotoma, and paracentral scotoma); iii) increased or normal intraocular pressure; and iv) at least three visible quadrants revealed by gonioscopy. NAION was diagnosed based on sudden loss of visual acuity, disc edema on fundus ophthalmoscopy during the acute stage, visual field defects consistent with NAION, erythrocyte sedimentation rate and reactive protein C levels within normal values with no signs of giant cell arteritis, at least 6 months after the acute phase. Healthy eyes without glaucoma, fundus disease or history of laser therapy or eye surgery were used as controls. These eyes had an intra-ocular pressure ≤21 mmHg and normal optic disc morphology and visual field. This study was conducted in accordance with the Declaration of Helsinki and with approval from the Ethics Committee of the First Affiliated Hospital of Xinxiang Medical University (Weihui, China). Written informed consent was obtained from all participants.

### Methods

**Figure 1.**
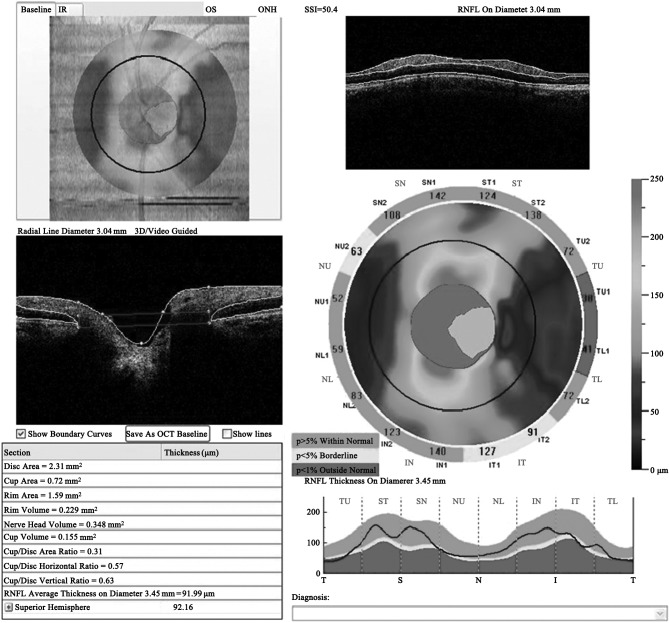
Diagram of procedure for optic nerve head (ONH) measurement by Fourier domain optical coherent tomography (OCT).

Tests for glaucoma were performed on all subjects. These tests included visual acuity examination, optometry, tonometry, slit lamp, gonioscopy, non-mydriatic fundus photography and Humphrey perimeter test. The parameters of optic nerve morphology, including optic disc and RNFL (diameter 3.45 mm) thickness, were obtained with RTVue FDOCT (4.0 version; Optovue Inc., Fremont, CA, USA) set in a program for measuring the optic nerve head (ONH). The parameters, including disc area, cup area, rim area, disc volume, cup volume, rim volume, C/D area ratio, C/D horizontal ratio, C/D vertical ratio and mean and localized RNFL thickness, were recorded (Fig. 1).

### Statistical analysis

Data analysis was performed using SPSS 16.0 (SPSS, Inc., Chicago, IL, USA). The χ^2^ test was used to compare the male to female gender ratio. One-way analysis of variance (ANOVA) and least significant difference (LSD) were used to compare age, SE or OCT parameters between two groups. The area under the receiver operating characteristic curve (AROC) was used to assess the diagnostic capacity of each parameter. The AROCs were calculated and analyzed using MedCalc 9.6.2.0 (MedCalc Software, Mariakerke, Belgium). P<0.05 was considered to indicate a statistically significant result.

## Results

### Characteristics of the subjects

Seventy-one subjects (38 men and 33 women) were evaluated. The mean age was 53.3±14.4 years. There were 26 cases of glaucoma (36.6%), 15 cases of NAION (21.1%) and 30 control cases (42.3%). There was no difference in gender ratio, age and SE among the groups (P>0.05). There were significant differences in the mean deviation (MD) and pattern standard deviation (PSD) of the Humphrey visual field analyzer among the three groups (P<0.01). The demographic data and optical characteristics of the patients and controls are listed in Table I.

### Comparison of optic nerve morphology parameters

The parameters of the optic disc and RNFL thickness measured in glaucomatous, NAION and normal control eyes are summarized in Table II. Using LSD analysis of the three groups, we observed that glaucomatous eyes had the largest cup area (1.438±0.714 mm^2^; P<0.05) and the smallest rim area (0.965±0.652 mm^2^; P<0.05). NAION eyes had the smallest cup area (0.493±0.344 mm^2^; P<0.05) and a rim area (1.255±0.294 mm^2^) that was larger than that of glaucomatous eyes, but comparable to that of control eyes (1.243±0.509 mm^2^; P>0.05). There was no significant difference in disc area between control eyes (2.194±0.618 mm^2^) and glaucomatous eyes (2.203±0.557 mm^2^; P>0.05) or NAION eyes (1.945±0.458 mm^2^; P>0.05). Glaucomatous eyes had the smallest ONH volume (0.195±0.168 mm^3^; P<0.05) and rim volume (0.103±0.089 mm^3^; P<0.05), and the largest cup volume (0.482±0.420 mm^3^; P<0.05). The NAION eyes had the smallest cup volume (0.083±0.073 mm^3^; P<0.05), but there was no significant difference in ONH volume (0.339±0.109 mm^3^) and rim volume (0.196±0.094 mm^3^) between NAION eyes and control eyes (P>0.05). The C/D ratio was the highest in glaucomatous eyes (C/D area ratio 0.591±0.256, C/D horizontal ratio 0.799±0.190 and C/D vertical ratio 0.764±0.196) and the lowest in NAION eyes (C/D area ratio 0.258±0.182, C/D horizontal ratio 0.445±0.302 and C/D vertical ratio 0.480±0.321).

The parameters of RNFL thickness in all regions of NAION eyes [except in the nasal lower (NL), inferior nasal (IN) and temporal lower (TL) regions] and glaucomatous eyes were lower than those in control eyes (P<0.05). The greatest loss of RNFL thickness was in the temporal upper (TU), superior temporal (ST) and TL regions of glaucomatous eyes and the superior nasal (SN) and nasal upper (NU) regions of NAION eyes.

### Diagnostic capabilities analysis of optic nerve morphology parameters in glaucoma and NAION

By analyzing the AROCs of glaucomatous eyes and control eyes, we observed that all parameters of the optic disc (with the exception of disc area) and RNFL thickness were significantly different between the two groups (P<0.05; Table III). Further analysis revealed that there was no difference in the AROC between the two parameters of the optic disc (P>0.05). However, the AROCs of RNFL thickness were higher than that of the optic disc (P<0.05). In detail, the AROCs of the superior (ST and SN), temporal (TU, TL and IT) and mean RNFL were significantly higher than those of nasal (NU and NL) RNFL (P<0.05).

By comparing the AROCs of NAION and control eyes, as shown in Table IV, we observed that there were no differences in any of the parameters of the optic disc. There was a significant difference only in the AROCs of the superior (ST and SN), nasal (NU) and inferior temporal (IT) RNFL (P<0.05).

We then compared the AROCs of the parameters in NAION and glaucomatous eyes. As shown in Table V, there were significant differences in the AROCs of the cup area, cup volume and C/D ratio (P<0.05). For the parameters of RNFL thickness, there were significant differences in the AROCs of the temporal (TU), nasal (NU) and mean RNFL thickness (P<0.05).

## Discussion

The typical characteristics of NAION include sudden vision loss, visual field defect and optic edema. Following the acute phase, optic disc pallor arises. Certain patients have no apparent vision loss and the visual field defects are seldom detected when patients receive professional diagnosis. The characteristics of NAION in the atrophic phase are similar to those of glaucomatous damage (1). Therefore, NAION patients are sometimes suspected of having glaucoma. In recent years, OCT has been used to detect the pathological changes of NAION. With the use of Stratus OCT, Contreras *et al* observed that the RNFL thickness (particularly the superior RNFL thickness) in NAION eyes was the highest in the acute phase, became thinner thereafter and reached its lowest level 6 months after the acute phase (10). There was a gradual reduction of the vertical rim area from the acute phase to the sixth month thereafter, whereas the C/D area ratio increased from the start of the acute phase. There was no change in the size of optic disc (11).

Due to the similarities between optic nerve morphology in NAION and glaucomatous eyes, comparison studies have been performed. Using HRT and Stratus OCT, Danesh-Meyer *et al* observed that with the same visual field defect, open-angle glaucomatous eyes had a larger cup area and volume, smaller rim area and rim volume, and higher RNFL thickness compared with NAION eyes (7). Horowitz *et al* used Stratus OCT to study NAION and glaucoma patients with a visual field defect less than a hemifield. The authors observed that there was no difference in RNFL thickness at corresponding areas of damage. However, the RNFL thickness in the non-damaged areas of glaucomatous eyes was less than that of NAION eyes and both were higher than that of control eyes (8). The results of the two studies were not consistent, likely due to the difference in the time at which the NAION patients were analyzed. The patients in the former study were examined at least 6 weeks after the acute phase, whereas the patients in the latter study were examined at >6 months after the acute phase. Contreras *et al* suggested that the RNFL in NAION patients was the thinnest at 6 months after the acute phase (10). In the present study, patients were examined at least 6 months after the acute phase. We observed that the RNFL thickness in NAION eyes was less than that of glaucomatous eyes. In addition, the acute ischemia of the optic nerve in NAION results in reduction of the function and death of retinal ganglia cells (RGCs) (12). The RNFL thickness in glaucomatous eyes was higher than expected, possibly since glaucoma does not kill RGCs, but reduces the function of their axons.

By comparing the parameters of NAION eyes and glaucomatous eyes to control eyes, we observed that the glaucomatous eyes had the largest cup area and C/D ratio and the smallest rim area. There was no difference in disc area among the three groups of eyes. The cup area and C/D ratio in NAION eyes were the smallest, but the rim area and disc area of the NAION eyes were similar to those of the control. These conclusions were consistent with the findings of previous studies (6,7). We also observed that the mean RNFL thickness, the thickness of the majority of RNFL regions in NAION eyes and all regions of RNFL in glaucomatous eyes were lower than those of the control. Among the three groups, the greatest loss of RNFL thickness was in the TU, ST and TL regions in glaucomatous eyes (P<0.05), and in the SN and NU regions of NAION eyes (P<0.05). Our data, together with previous studies (10,13,14), demonstrate that the temporal RNFL in glaucomatous eyes and the superior RNFL in NAION eyes are the most vulnerable to damage.

We observed that all the parameters were useful for the diagnosis of glaucoma, with the exception of disc area, and the parameters of RNFL were the most valuable. Previous studies suggest that the AROCs of mean, superior and inferior RNFL thickness in glaucomatous eyes were larger than those of control eyes (13). After investigating 88 cases of glaucoma, Lisboa *et al* (14) observed that among the parameters obtained from SD-OCT measurement, the diagnostic capacity (from excellent to poor) was ST, mean and IT RNFL thickness. Among the parameters of optic disc morphology obtained using HRT-III, the diagnostic capacity (from excellent to poor) was rim area, rim volume and linear C/D ratio. The diagnostic capacity of superior temporal RNFL thickness measured by OCT was more accurate than that of the rim area measured by HRT (0.88 vs. 0.72; P=0.008). Our results also suggest that RNFL thickness has a more accurate diagnostic capacity.

In the current study, we observed that the regions that were useful in the diagnosis of NAION were ST, SN, NU and IT. The parameters that were useful for distinguishing NAION and glaucoma were cup area, cup volume, C/D ratio (area, horizontal and vertical) and RNFL thickness (TU, NU and mean). Previous studies suggest that NAION eyes have a small optic disc, optic cup and C/D ratio (2,3,6). The changes in superior RNFL were more evident in NAION eyes. Thus, these changes are valuable in diagnosis.

In addition, in the current study, we used a new generation of FDOCT, which has faster scanning speed and improved sensitivity. The parameters collected using this new tool were more accurate than those from the previous generation. However, we only collected data from patients 6 months after the NAION acute phase. Information concerning dynamic changes in the acute phase is lacking. Further studies to examine the characteristic changes of NAION diseases are required.

In conclusion, by using FDOCT, we observed that compared with the control, the glaucomatous eyes had a larger cup area, smaller rim area and larger C/D ratio. NAION eyes had a smaller cup area and C/D ratio. The RNFL thickness in glaucomatous eyes was the most reduced in the ST and IT regions, and in NAION eyes in the SN region. Thus, the characteristics of cup area, cup volume, C/D ratio and RNFL thickness may distinguish between these two similar diseases.

## Figures and Tables

**Table I.** Demographics and optic characteristics of glaucoma, NAION and normal subjects.

Variable	Glaucoma	NAION	Control	F-statistic	P-value
Age, mean ± SD (years)	50.7±19.3	57.5±9.10	51.4±16.1	0.620	0.541
Gender (n, % male)	14, 53.8%	7, 46.7%	17, 56.7%	0.404	0.817
SE (D)	2.05±0.71	1.94±0.81	2.40±1.25	0.461	0.645
MD (dB)	10.2±7.8	9.6±7.1	1.2±1.3	20.786	0.000
PSD (dB)	9.7±4.2	7.9±4.2	2.8±0.9	34.351	0.000

NAION, non-arteritic anterior ischemic optic neuropathy; SE, spherical equivalent; MD, mean deviation of the Humphrey visual field analyzer; PSD, pattern standard deviation of the Humphrey visual field analyzer.

**Table II.** Comparison of Fourier domain optical coherence tomography (FDOCT) parameters in glaucomatous, NAION and normal eyes.

Variable	NAION	Glaucoma	Control	F-statistic	P-value
ONH					
Disc-area (mm^2^)	1.945±0.458	2.203±0.557	2.194±0.618	3.068	0.059
Cup-area (mm^2^)	0.493±0.344	1.438±0.714	0.952±0.605	9.967	0.000
Rim-area (mm^2^)	1.255±0.294	0.965±0.652	1.243±0.509	3.312	0.041
Rim-volume (mm^3^)	0.196±0.094	0.103±0.089	0.175±0.119	7.714	0.001
Nerve head-volume (mm^3^)	0.339±0.109	0.195±0.168	0.318±0.219	0.345	0.002
Cup-volume (mm^3^)	0.083±0.073	0.482±0.420	0.287±0.257	5.793	0.004
C/D area	0.258±0.182	0.591±0.256	0.416±0.239	9.225	0.000
C/D horizontal	0.445±0.302	0.799±0.190	0.689±0.298	5.844	0.004
C/D vertical	0.480±0.321	0.764±0.196	0.605±0.256	8.672	0.000
RNFL (*μ*m)					
Mean	55.75±26.16	88.79±17.37	113.80±10.25	54.990	0.000
TU	108.62±32.84	64.89±19.22	91.58±14.42	37.756	0.000
ST	113.38±32.57	105.85±23.96	144.53±16.12	46.954	0.000
SN	79.38±17.86	100.66±19.62	129.34±16.75	40.774	0.000
NU	48.50±11.40	68.16±16.15	77.52±13.11	10.581	0.000
NL	81.00±21.55	69.50±14.13	80.18±14.81	7.926	0.001
IN	131.50±18.43	119.57±32.48	143.67±21.98	10.331	0.000
IT	113.62±25.42	118.53±32.57	159.95±22.45	30.992	0.000
TL	72.55±17.87	63.67±14.40	83.33±12.09	29.633	0.000

NAION, non-arteritic anterior ischemic neuropathy; ONH, optic nerve head; C/D, cup-to-disc ratio; RNFL, retinal nerve fiber layer; TU, temporal upper; ST, superior temporal; SN, superior nasal; NU, nasal upper; NL, nasal lower; IN, inferior nasal; IT, inferior temporal; TL, temporal lower.

**Table III.** AROCs for ONH and RNFL thickness in glaucomatous eyes compared with control eyes.

Variable	AROC	SE	95% CI	P-value
ONH				
Disc-area	0.604	0.054	0.498–0.710	0.056
Cup-area	0.693	0.048	0.598–0.788	0.000
Rim-area	0.664	0.051	0.565–0.763	0.003
Rim-volume	0.700	0.049	0.605–0.795	0.000
Nerve head-volume	0.691	0.049	0.595–0.787	0.000
Cup-volume	0.634	0.051	0.533–0.735	0.014
C/D area	0.698	0.048	0.604–0.792	0.000
C/D horizontal	0.628	0.053	0.524–0.732	0.019
C/D vertical	0.706	0.048	0.612–0.800	0.000
pRNFL				
Mean	0.891	0.029	0.834–0.949	0.000
TU	0.876	0.033	0.810–0.941	0.000
ST	0.904	0.029	0.847–0.961	0.000
SN	0.868	0.034	0.802–0.933	0.000
NU	0.660	0.050	0.561–0.758	0.003
NL	0.692	0.049	0.596–0.788	0.000
IN	0.728	0.046	0.637–0.819	0.000
IT	0.837	0.036	0.766–0.908	0.000
TL	0.867	0.034	0.801–0.933	0.000

AROC, area under the receiver operator characteristic curve; ONH, optic nerve head; RNFL, retinal nerve fiber layer; CI, confidence interval; C/D, cup-to-disc ratio; pRNFL, peripapillary retinal nerve fiber layer; TU, temporal upper; ST, superior temporal; SN, superior nasal; NU, nasal upper; NL, nasal lower; IN, inferior nasal; IT, inferior temporal; TL, temporal lower.

**Table IV.** AROCs for ONH and RNFL thickness in NAION eyes compared with control eyes.

Variable	AROC	SE	95% CI	P-value
ONH				
Disc-area	0.750	0.107	0.539–0.961	0.099
Cup-area	0.750	0.081	0.591–0.909	0.099
Rim-area	0.508	0.105	0.302–0.713	0.960
Rim-volume	0.605	0.144	0.323–0.887	0.488
Nerve head-volume	0.585	0.105	0.379–0.791	0.574
Cup-volume	0.737	0.086	0.570–0.905	0.117
C/D area	0.700	0.098	0.508–0.892	0.186
C/D horizontal	0.770	0.078	0.618–0.922	0.074
C/D vertical	0.667	0.103	0.466–0.869	0.269
pRNFL				
Mean	0.900	0.057	0.789–1.011	0.900
TU	0.295	0.207	−0.110–0.700	0.176
ST	0.822	0.094	0.639–1.006	0.033
SN	0.990	0.012	0.966–1.014	0.001
NU	0.990	0.012	0.967–1.013	0.001
NL	0.495	0.193	0.118–0.872	0.974
IN	0.655	0.108	0.443–0.867	0.306
IT	0.932	0.057	0.820–1.045	0.004
TL	0.680	0.151	0.383–0.977	0.234

AROC, area under the receiver operator characteristic curve; ONH, optic nerve head; RNFL, retinal nerve fiber layer; NAION, non-arteritic anterior ischemic neuropathy; CI, confidence interval; C/D, cup-to-disc ratio; pRNFL, peripapillary retinal nerve fiber layer; TU, temporal upper; ST, superior temporal; SN, superior nasal; NU, nasal upper; NL, nasal lower; IN, inferior nasal; IT, inferior temporal; TL, temporal lower.

**Table V.** AROCs for ONH and RNFL thickness in NAION eyes compared with glaucomatous eyes.

Variable	AROC	SE	95% CI	P-value
ONH				
Disc-area	0.778	0.087	0.618–0.979	0.064
Cup-area	0.881	0.051	0.781–0.980	0.011
Rim-area	0.685	0.073	0.542–0.827	0.218
Rim-volume	0.792	0.092	0.613–0.972	0.051
Nerve head-volume	0.762	0.077	0.610–0.913	0.081
Cup-volume	0.833	0.067	0.702–0.963	0.026
C/D area	0.850	0.061	0.730–0.970	0.019
C/D horizontal	0.887	0.049	0.790–0.984	0.010
C/D vertical	0.827	0.064	0.701–0.953	0.029
pRNFL				
Mean	0.823	0.099	0.629–1.018	0.031
TU	0.867	0.109	0.665–1.080	0.014
ST	0.606	0.188	0.238–0.974	0.480
SN	0.775	0.090	0.599–0.951	0.066
NU	0.844	0.060	0.727–0.962	0.022
NL	0.675	0.168	0.346–1.004	0.243
IN	0.619	0.088	0.447–0.791	0.426
IT	0.558	0.124	0.316–0.800	0.700
TL	0.642	0.165	0.319–0.965	0.342

AROC, area under the receiver operator characteristic curve; ONH, optic nerve head; RNFL, retinal nerve fiber layer; NAION, non-arteritic anterior ischemic neuropathy; CI, confidence interval; C/D, cup-to-disc ratio; pRNFL, peripapillary RNFL; TU, temporal upper; ST, superior temporal; SN, superior nasal; NU, nasal upper; NL, nasal lower; IN, inferior nasal; IT, inferior temporal; TL, temporal lower.
